# Supplementary material for: Sensitivity Analysis of Cardiac Alternans and Tachyarrhythmia to Ion Channel Conductance Using Population Modeling
Source: Bioengineering (Basel). 2022 Nov 1;9(11):628. doi: 10.3390/bioengineering9110628 (PMC9687149; doi:10.3390/bioengineering9110628)
Supplement: Supplementary file 1 [file bioengineering-09-00628-s001.zip › bioengineering-1913811-supplementary.pdf]

## Supplementary Materials

## 1 Expression of ion channel currents [12]

Fast $Na^+$ current ($I_{Na}$):

$$I_{Na} = G_{Na} m^3 hj (V_m - E_{Na})$$ **Equation 1**

L-type $Ca^{2+}$ current ($I_{CaL}$):

$$I_{CaL} = G_{CaL} d f f_{Ca} 4 \frac{V_m F^2 Ca_i e^{2V_m F/RT} - 0.341 Ca_o}{e^{2V_m F/RT} - 1}$$ **Equation 2**

Transient outward current ($I_{to}$):

$$I_{to} = G_{to} rs (V_m - E_K)$$ **Equation 3**

Slow delayed rectifer current ($I_{Ks}$):

$$I_{Ks} = G_{Ks} x_s^2 (V_m - E_{Ks})$$ **Equation 4**

Rapid delayed rectifier current ($I_{Kr}$):

$$I_{Kr} = G_{Kr} \sqrt{\frac{K_o}{5.4}} x_{r1} x_{r2} (V_m - E_K)$$ **Equation 5**

Inward rectifier $K^+$ current ($I_{K1}$):

$$I_{K1} = G_{K1} \sqrt{\frac{K_o}{5.4}} x_{K1\infty} (V_m - E_K)$$ **Equation 6**

Plateau $Ca^+$ current

$$I_{pCa} = G_{pCa} \frac{Ca_i}{K_{pCa} + Ca_i}$$ **Equation 7**

Plateau $K^+$ current

$$I_{pK} = G_{pK} \frac{V_m - E_K}{1 + e^{(25 - V_m)/5.98}}$$ **Equation 8**

Background sodium and calcium leakage current

$$I_{bNa} = G_{bNa}(V_m - E_{Na})$$ **Equation 9**

$$I_{bCa} = G_{bCa}(V_m - E_{Ca})$$ **Equation 10**

## Reference

[12]. Ten Tusscher, K.H.W.J., A model for human ventricular tissue. *AJP Hear. Circ. Physiol.* 2004; Volume 286, Issue 4, pp. H1573–H1589.

## Supplementary Figures

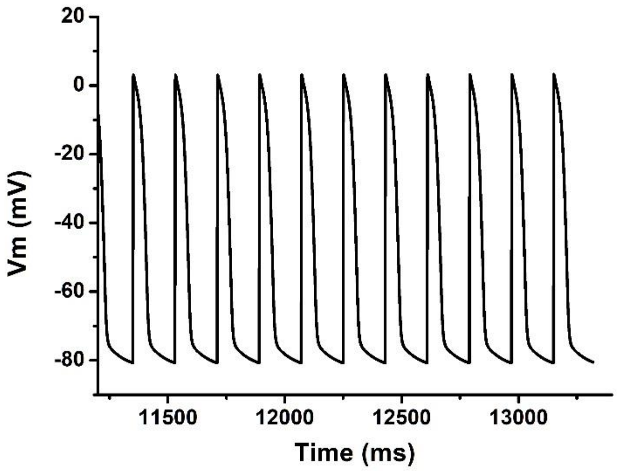


**Supplementary Figure S 1**. Representative action potential trace under the no alternans condition

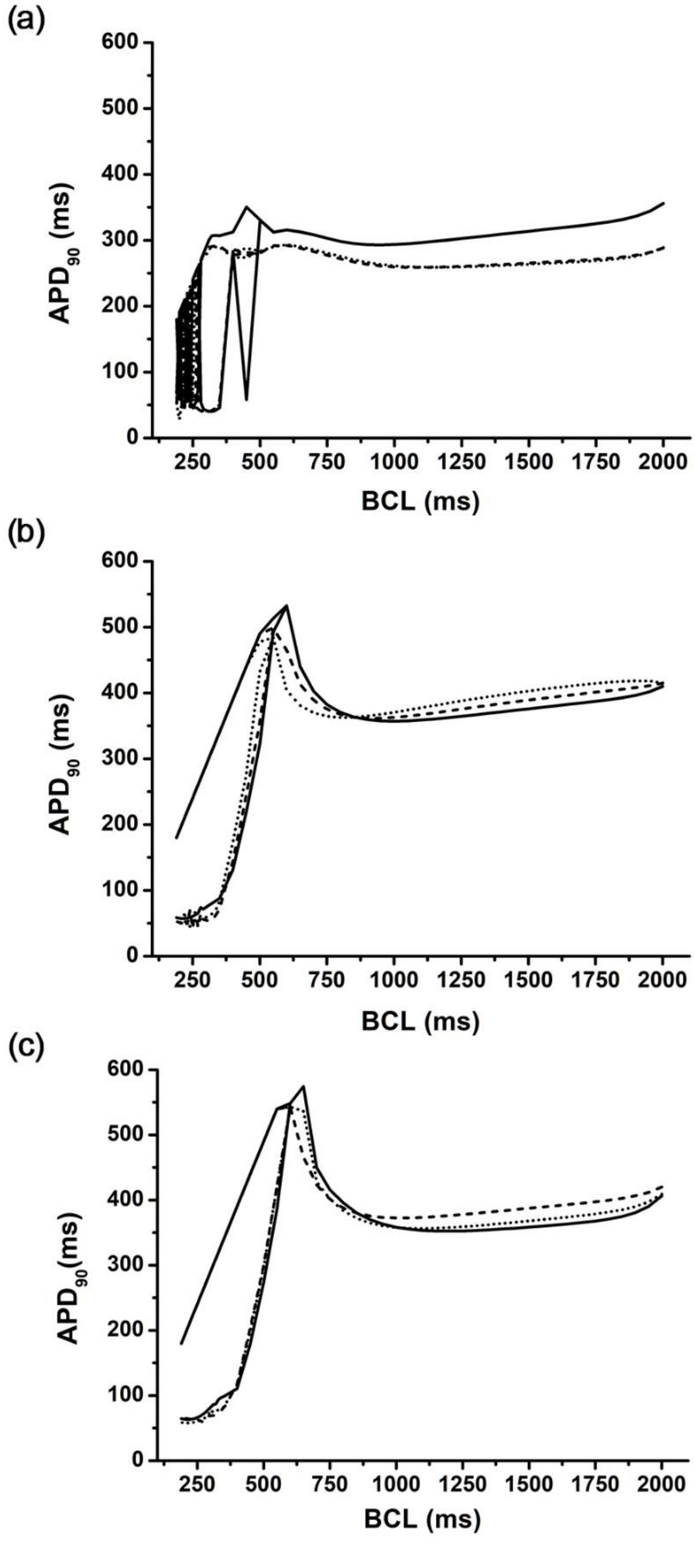


**Supplementary Figure S 2.** APDr curve in the cases that APD alternans occur under the resting cycle length condition; (a), AOCL = 450 ms; (b), AOCL= 500ms; (c), AOCL =550 ms

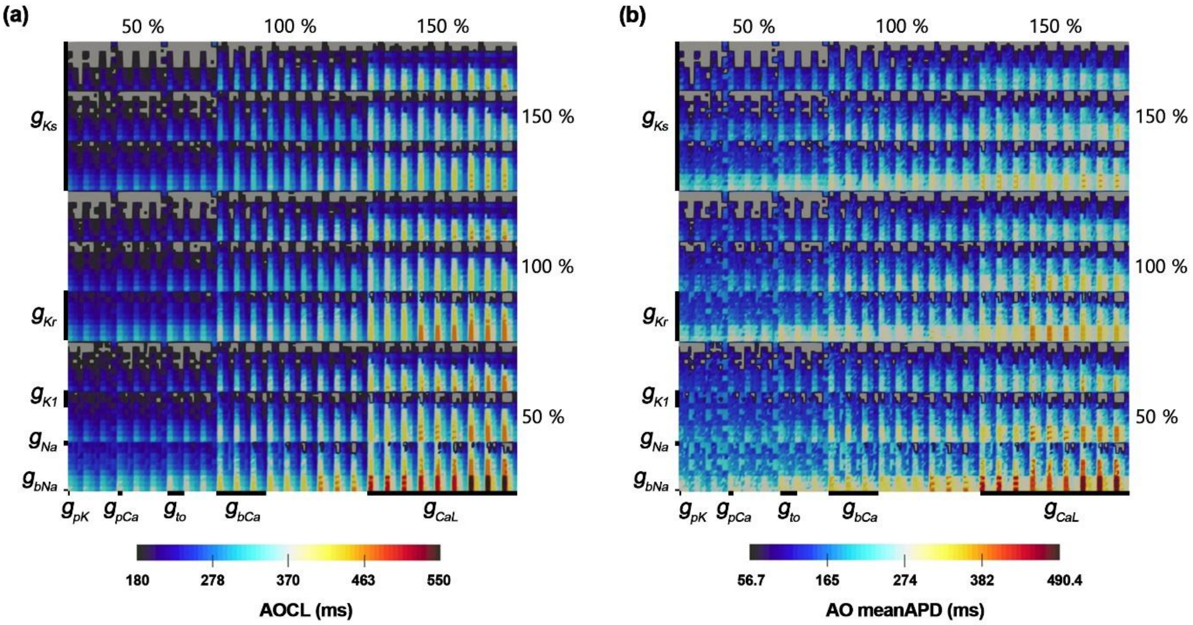


**Supplementary Figure S 3.** Unoptimized population maps of AOCL (a) and alternans onset mean APD (b); AOCL, alternans onset cycle length; APD, action potential duration; Gray parts represent no APD alternans scenarios.
